# Heavy metal pollution from a shooting range revealed by honeybees

**DOI:** 10.1007/s10661-025-14807-8

**Published:** 2025-11-29

**Authors:** Vivian Leuenberger, Juanita Rausch, David Jaramillo, Christoph Neururer, Bernard Grobéty

**Affiliations:** 1 Department of Geosciences, University of Fribourg, Chemin du Musée 7, Fribourg, 1700 Switzerland; 2 Particle Vision GmbH, Passage du Cardinal 13B, Fribourg, 1700 Switzerland

**Keywords:** Honey bees, Aerosol, Biomonitoring, Heavy metal, Computer controlled scanning electron microscopy (CCSEM)

## Abstract

Honey bees (*Apis mellifera* L.) have been established as environmental monitors to assess the aerosol contamination of the environment in the vicinity of beehives. During their wide-ranging foraging trips, these hymenopterans catch particles in flight and while collecting nectar, pollen, or water. This study demonstrates that honeybees can be employed not only to detect major particulate matter emitters, such as mines or industrial plants, but also smaller, localized aerosol sources, such as shooting ranges. During background monitoring of particulate matter pollution, worker bees were collected from hives in rural areas of the canton and the city of Fribourg (Switzerland). The head, wings, and hind legs of the bees were investigated with Scanning Electron Microscopy coupled with X-ray spectroscopy (SEM–EDX). The analyzed particles reflect the vigorous dairy farming activity in the region (home of Gruyère cheese-making), but particles from one beehive were very exotic and typical of gunshot residues. Indeed, a shooting range was within the foraging range of the corresponding beehive. Bees, therefore, could be an ideal tool not only for monitoring significant aerosol sources but also for monitoring small ones.

## Introduction

Airborne particulate matter contributes significantly to PM10 pollution. PM10 refers to particles with diameters equal to or less than 10 µm. Particles can either be of natural or anthropogenic origin. They are divided into primary particles, i.e., emitted directly from a source (e.g., mineral dust), or secondary particles formed by the condensation of precursor gaseous compounds (e.g., ammonium nitrate). Important natural sources include rock and soil erosion, salt particles emitted from the sea, volcanic eruptions, and wildfires (Buseck & Pósfai, 1999; Gieré & Querol, 2010). Industry, agriculture, and traffic are the most important anthropogenic sources of PM 10 (Grobety et al., 2010). Epidemiological studies have demonstrated clear relationships between gaseous pollutants and particulate matter with adverse health outcomes, including mortality due to cardiovascular and respiratory diseases (Shiraiwa et al., 2017).

The nature and concentration of particles suspended in the atmosphere are typically measured through either active or passive collection of air samples at specific locations (Vincent, 2007). The origin of the air volume and, therefore, of the aerosol sampled will depend on the movements of the air masses, e.g., direction, changes of direction, speed, and the ground touched by the air masses during transport. To identify possible particle sources, the latter factors are used to calculate back trajectories for the sampled air masses (Cohen et al., 2015).

Biomonitoring is a cost-effective and valuable tool for assessing atmospheric aerosol pollution. Organisms such as lichens (Paoli & Loppi, 2008), mosses (Bargagli, 2016), tree bark (Chaparro et al., 2020), and bees (Negri, 2015) accumulate and concentrate airborne contaminants, providing a record of environmental quality over time. Bees are foraging within a defined area; they are thereby exposed to pollutants present in the atmosphere, soil, vegetation, and water, which are incorporated in their bodies. They ingest contaminated nectar, pollen, and water or inhale gaseous pollutants. Contaminated airborne particles may adhere to their hairy exterior. Bees and bee products have, therefore, been used as environmental pollution indicators (Badiou-Bénéteau et al., 2013; Bargańska et al., 2016; Conti & Botre, 2001; Crane, 1984; Cunningham et al., 2022; Jones, 1987; Raeymaekers, 2006; Satta et al., 2012; Smith et al., 2019; Steen et al., 2012). Researchers analysed their bodies or apiary products, such as honey and wax. The concentration of pollutants within the bees’ bodies increases as they chemically clean the raw honey of these pollutants during processing. The latter are accumulated in their tissues (Roman & Demenczuk, 2003). Particulate matter pollution was evaluated by analysing the particles found on the bee’s body by Computer Controlled Scanning Electron Microscopy (CCSEM) (Negri, 2015; Pellecchia et al., 2023). Aerodynamic factors and the grooming behavior of bees are responsible for the preferential body parts on which particles are found, e.g., the costal margin of the fore wings, the medial plane of the head, the inner surface of the hind legs, and all areas containing secreted wax (Negri et al., 2015). During their flight, bees collide with particles that attach to their surface. They become mobile aerosol collectors. In this paper, we present data on particles collected on bees during a general monitoring of the background aerosols found in the area of Fribourg (Switzerland). The results reveal, in contrast to previous particulate matter monitoring with bees, which targeted rather large emitters such as mining areas (Negri et al., 2015), cement plant operations (Pellecchia & Negri, 2018), cities (Capitani et al., 2021; Pellecchia et al., 2023), in general (Papa et al., 2025) (Wang et al., 2013), a small, rather special emission source e.g. a shooting range emitting gunshot residue particles.

The bees sampled in all apiaries belong to the most common bee species worldwide, e.g., Western honeybee (*Apis mellifera *L*.*). The only bees leaving the beehive are the worker bees during foraging and orientation flights. Depending on the season, up to 100′000 worker bees may live in one colony (Winston, 1987). Anatomically, the 10–11 mm long bee is divided into three parts: head, thorax, and abdomen. They have evolved many specialized structures to collect and transport pollen. Pollen is actively trapped by specialized forager bees (Danner et al., 2016) in the setae (hair-like structures) or caught via electrostatic forces. Bees may inadvertently collect particles other than pollen. It is essential to consider that bees can clean themselves through self-cleaning and social grooming. With the middle legs, they clean the thoracic hairs, with the antenna cleaner on the foreleg, e.g., a curved notch and associated spur, they pull and brush the antennae, with brushes on the hind legs, they clean middle leg spines and the brushes between themselves (Schönitzer & Renner, 1984). Self-cleaning starts with a grooming dance (Milum, 1947), which may initiate social grooming in "temporarily specialized" groomer bees, which often groom several other bees repeatedly over several days (Kolmes, 1989). They preferentially clean those body parts that are out of reach during self-cleaning. The grooming behavior removes dust and pollen from the wing bases and petiolus and realigns the body hairs (Božič & Valentinčič, 1995). That means that such cleaning activities may bias the number and types of particles found on the bees. Particles and components thereof may be found in apiary products, such as wax, propolis, and even in honey (Bogdanov, 2006; Negri et al., 2015; Yatim & Azman, 2021). Weather and other environmental factors influence the foraging. Bees can fly during winter, but generally do not forage at temperatures below 12–14 °C (Winston, 1987).

## Methods

### Sites and sampling periods

**Fig. 1:**
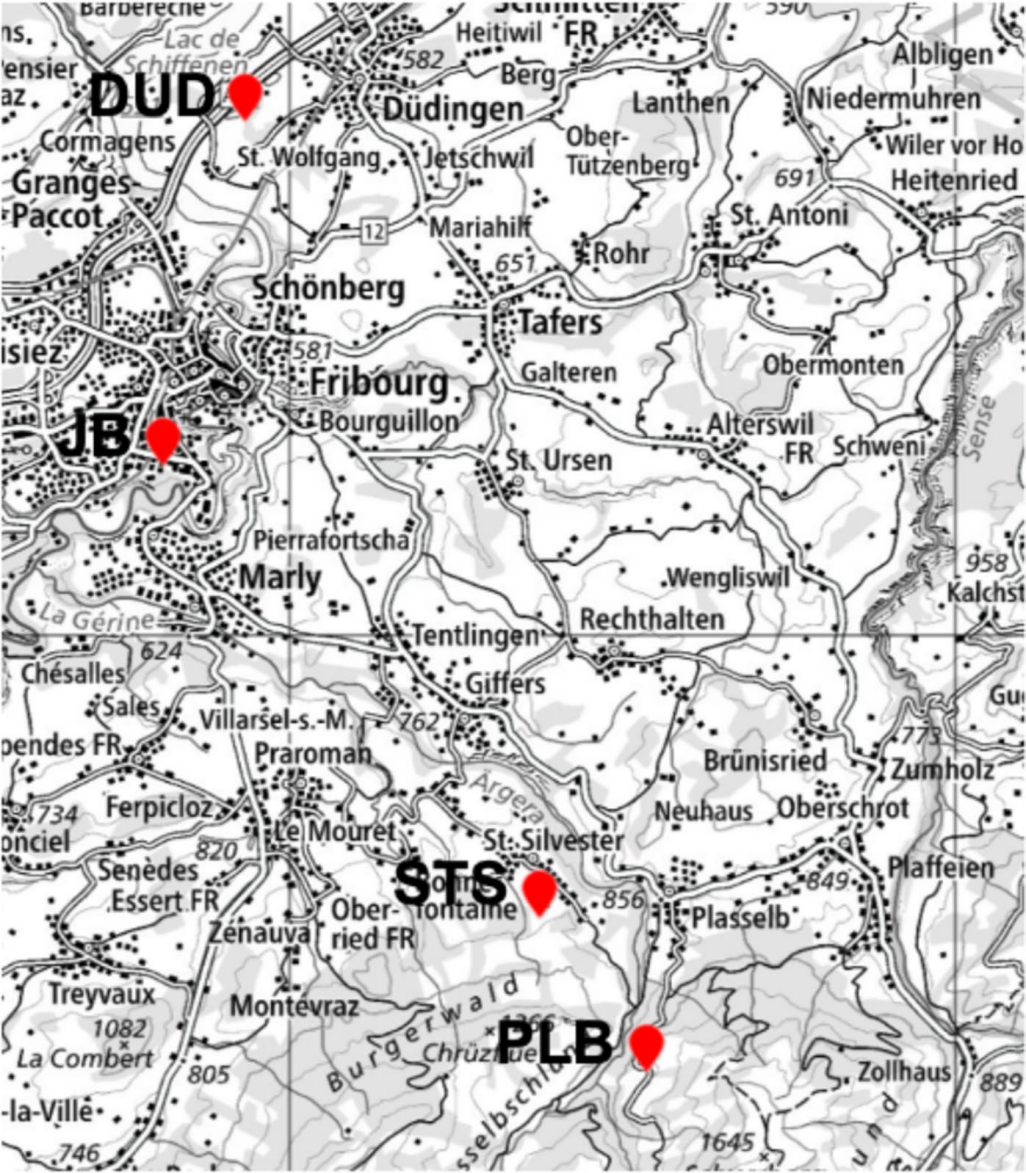
Map with the localities (red dot-arrows) of the sampled apiaries. (https://www.swisstopo.admin.ch)

Four apiaries in the canton of Fribourg, Switzerland (Fig. 1), located in different environments, were chosen (Table 1), the first close to the Swiss high way A2 and the track of the Swiss railway (SBB) in Düdingen (DUD, Swiss coordinates (*CH1903/LV95): 2′579′198, 1′187′717*), two rural sites Plasselb (PLB, *2′585′195, 1′173′485)*, and St. Silvester (STS, *2′583′596, 1′175′668))* and the last one in an urban site e.g. in the city of Fribourg (JB, *2′578′423, 1′182′407*). The PLB site is far away from settlements. It is next to a road, but with very little traffic (about 10 cars per day). The anthropogenic near-field influence is negligible, except for emissions from a quartz sandstone quarry located 500 m away. The STS beehive is situated in a rural environment, surrounded by a few settlements and forests, with meadows nearby. A shooting range lies within the bees’ harvesting territory, which we were not aware of at the beginning of the campaign. The JB beehive is located on the outskirts of the city. It is situated in the university’s botanical garden (“Jardin Botanique”), which is separated from the nearest roads by buildings but open to the Sarine gorge, which has wood-covered flanks. Both the botanical garden and the gorge exhibit a high level of plant diversity.

**Table 1:** Bee collection and filter sampling parameters

Campaign	Site	Bee sampling	Conditions and remarks	Filter sampling	Conditions and remarks	Volume of sample [m^3^]
A	Plasselb (PLB)	18.09.2019	cloudy, 15 °C	30.09.2019	cloudy, strong wind, 16 °C	1.74
	Fribourg (FR)	19.09.2019	sunny, windy, 25 °C	07.10.2019	cloudy, dry, 17 °C, road works in proximity	1.08
	Düdingen (DUD)	19.09.2019	sunny, windy, 25 °C, gravel road; lot of sus- pended	29.09.2019	sunny, windy, 18 °C	1.39
	St. Silvester (STS)	18.09.2019	cloudy, 15 °C	03.10.2019	sunny, cold, 10 °C,	1.72
B	St. Silvester before shooting	29.05.2020	sunny, windy, 20 °C	05.06.2020	light rain, cloudy, 10 °C	1.60
	St. Silvester after shooting	13.06.2020	sunny, low wind, 20 °C, honey extraction shortly before	13.06.2020	sunny, low wind, 20 °C, slurry taste in the air from the nearby farm	0.98

Four bees per beehive were collected during the first campaign (A) in September 2019. As a result of the campaign A, the STS apiary was sampled a second time (one bee for the pre- and post-sampling) in June 2020 (campaign B) during a week of shooting training (Table 1). Active filter-based sampling of the aerosol in the surroundings of the apiaries (campaign A: one filter per beehive) with the prevailing winds blowing mainly from a southwesterly direction, less frequently from a northeasterly direction (the sampling at the STS beehive was repeated in early summer 2020 (campaign B: one filter each in the pre- and postshooting periods),. The samples were taken on polycarbonate filters (0.4 mm pore radius, 25 mm diameter) using a homemade pump and a sampling head developed at the Swiss Federal Laboratories for Material Science and Technology EMPA (Lorenzo et al., 2006). The airflow was set at 4 L/min, and depending on the site and the respective average aerosol concentration, the air was pumped for between 4.25 and 8.5 h.

#### Bee sampling 

The bee sampling was conducted with the owner’s permission. The bees have been caught alive directly at the entrances of the beehive. They were killed by suffocation in hermetic vessels, in which they were kept for one week after their death at room temperature and then prepared for the SEM and EDS analyses. No endangered or protected species are involved in this research.

#### SEM–EDS

**Fig. 2:**
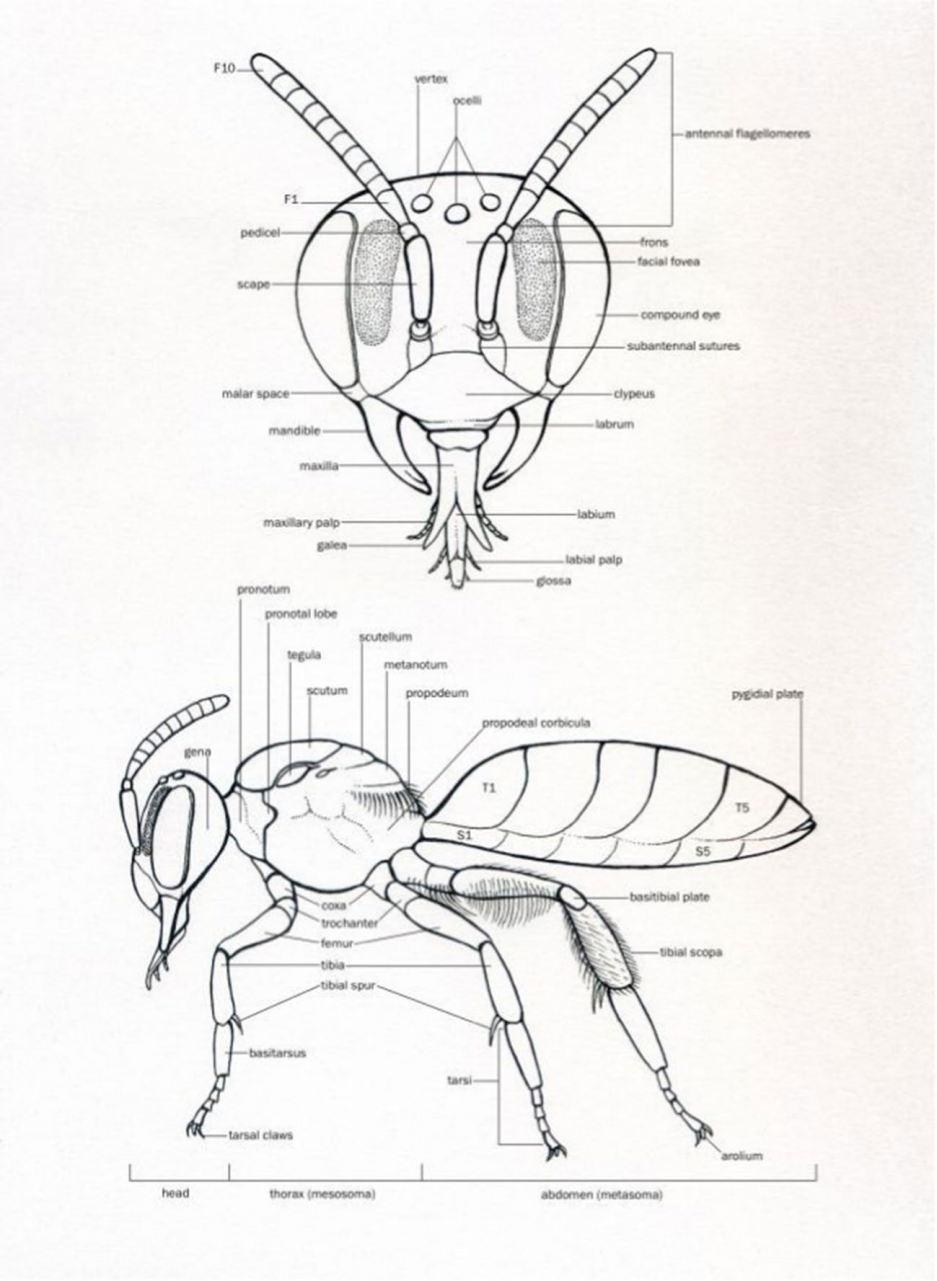
External structure of an adult worker bee. (https://ch.pinterest.com/pin/6614730698478533/)

The bees have always been positioned in the same way on a double-sided adhesive spectro-tab, so that at least the head, the right wings, the three right legs, and the antenna are visible (Fig. 2). Studies by Negri et al. (2015) have shown that particles are concentrated along the medial plane of the head, the costal margin of the forewings, and the inner surfaces of the hind legs. The bees were analysed in this order. To remove humidity, the bee samples were placed overnight in a high-vacuum chamber of a carbon vaporizer (Balzer CED 030, 10^–6^ mbar), before being coated with a 50 nm carbon layer. To better capture pollen morphology, a bee from the DUD apiary was gold-coated. For the particle filters, circular areas (8 mm in diameter) were punched out and carbon-coated.

The bees and filters have been analyzed using Scanning Electron Microscopy (SEM, FEI XL Sirion FEG, operated at 20 kV) equipped with an Energy Dispersive X-ray Spectrometry system (EDS, Oxford AZtec Advanced) and two BSE detectors: a Centaurus and a 4-quadrant semiconductor backscatter detector. For campaign B samples, the latter has been used, thereby avoiding the shadowing effect of the Centaurus detector and increasing both the depth of focus and resolution. The detection of the particles on the bee bodies was done "manually," e.g., by visual inspection of the backscatter scatter electron (BSE) images. Each particle was analyzed by point analysis. A "ZAF" correction (PAP version of the f(rz) formalism (Pouchou & Pichoir, 1985)) was applied automatically by the Aztec software on the raw data to calculate the elemental concentrations. Particle quantification was performed using a set of factory-delivered standard measurements. The ZAF procedure is only valid for homogeneous samples with smooth surfaces perpendicular to the beam and considerably thicker than the excitation volume diameter (Conconi et al., 2023). Neither is the case for the particles in our case. But several authors (Kandler et al., 2018; Meier et al., 2018) have shown that for particles larger than 1 micron in diameter, the relative 1- σ standard deviation for corrected concentrations of major elements is in the range of 2%, whereas for minor elements the deviation is considerably larger (Kandler et al., 2018). As only particles with diameters ≥ 1 µm were considered in the further processing of the data, the ZAF-corrected concentration values were retained without applying more sophisticated correction procedures. Each bee’s body is scanned following the same sequence, e.g., from the antenna to the eye, the front, middle, and hind leg, and to the wings. Approximately 10 BSE images per bee were scanned, each containing 20–150 particles. Magnification, beam size, and other parameters were adjusted for each EDS analysis.

**Fig. 3:**
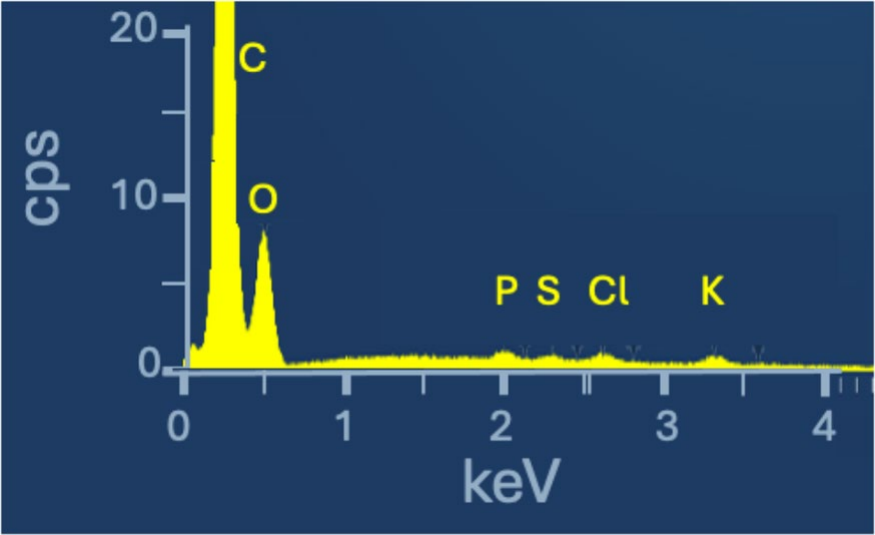
The EDS spectrum of the bee’s exoskeleton

Most of the particles on the bees are in the 1–50 μm size range. The smallest detectable particles are in the nano range. The compositions of all particle sizes ≥ 1 µm were considered, but not every particle image was recorded due to high particle number. Many particles are unstable under the electron beam. "Blank" spectra (Fig. 3) of the bee body have been obtained. The latter is covered by a thin layer (0.1–0.4 µm) of epicuticular waxes (Hepburn et al., 1991) and the particles are either entrapped inside the wax or simply adhere to the surface. The spectra contain major C and O, as well as minor P, S, and K, and Al, all elements, except Al, expected for arthropod exoskeletons, which are composed of chitin, proteins, and melanin (Draczynski, 2008). The aluminum peak is attributed to stray X-rays from the Centaurus BSE detector, which was used for most of the analyses. Therefore, the latter elements appearing in particle spectra must be taken with caution. After campaign A, a total of 1592 particles on the bees and 8934 particles on the filters, after campaign B a total of 464 particles on the two bees and 8895 particles on the two filters were measured (note the much higher average number concentration on both bees and filters compared to campaign A).

CT-analyses of the bees were attempted, but results show limited usefulness.

The filters have been analyzed using an automated procedure, called Computer Controlled Scanning Electron Microscopy (CCSEM) of single particles (Meier et al., 2018), a tool also implemented in AZTec. The particles are automatically recognized based on their contrast in BSE images, and an EDS spectrum is obtained. This automatic procedure is not possible for bee bodies because their hairs and cuticles are affected by charging, resulting in the same contrast as for particles. Depending on the particle occupancy on a filter, the size of the analyzing area was adjusted; for example, the scanning area ranges from 0.1 to 0.4 mm2, allowing for the measurement of approximately 1000–5000 particles.

### Honey analysis

Honey collected from three beehives (JB, STS, PLB) over 6 months prior to the start of campaign A was analyzed by solution inductively coupled plasma mass spectrometry (ICP-MS). A PerkinElmer Elan DRC Quadrupole ICP-MS with a nebulizer was used to analyze the digested solutions. The sample sizes were 200 g, and the following elements were analysed: Pb, Hg, Zn, Cu, Sb, and Ba.

### Particle classification

All analyses were corrected using the PAP procedure and normalized to 100 wt% (Meier et al., 2018). Before classification, oxygen, carbon, and elements with concentrations of less than 5wt% have been subtracted, and the concentrations of the remaining elements renormalized to 100%. Nineteen elements other than carbon and oxygen (Na, Mg, Al, Si, P, S, Cl, K, Ca, Ti, Cr, Mn, Fe, Ni, Cu, Zn, Ba, Te, and Pb) have been identified, considering all measured particle compositions, and were taken for the classification. The classification procedure was performed using the automated two-stage Particle Classification routine, PACLA (Meier et al., 2018). With PACLA, each element combination found in the measurements represents a subclass; for example, SiO_2_ (quartz) would be attributed to the subclass characterized by a single element, Si, CaCO_3_ (Calcite) to the subclass Ca, Fe_2_O_3_ to the subclass Fe, and NaAlSi_3_O_8_ to the subclass (Na, Al, Si), etc. This classification scheme led to 22 subclasses, which have been, after taking into consideration also the particle morphology, grouped into seven main classes (Table 2): pollen, minerals, salt (mainly NaCl), metals (including gun shot residue particles), sulfates (including elemental sulfur and nitrates), soot, and unknowns. The GSR particles were included in the metal group. Some "subclasses" are combinations of already existing subclasses and are most likely the result of particle mixtures.

**Table 2:** Classification scheme of particles found on bees and filters. One of the elements in bold letters must be present

Class	Elements present in the subclasses
Pollen	**P,** K, minor S, Cl, Fe
Soot	**C**, S
Mineral	**Si**, Al, K, Ca, Na, Mg, Fe, **Ca**
Salt	Na, **Cl**, K
Metal	Ti, Cr, Cu, Zn,Fe, Ni, Co
Sulfate + nitrates	**S**, N, Na, K, Mg, Ca
Gunshot residue (GSR)	Cu, Zn, Sn, Ba, Hg, Pb
Unknown	Al

## Results

### Particles

Particles from all main groups, except the gunshot residue particles, were found on every bee and filter collected in each of the four sites, but in different proportions.

### Pollen

Pollen is chemically complex to distinguish from the bee’s body, as both contain mainly C, N, and O. Carbon is also present in the carbon coating. The main compounds in bee pollen are organic, including proteins, lipids, sugars, fibre, carbohydrates, amino acids, phenolic compounds, and vitamins (Campos et al., 2008). Minor inorganic mineral elements include iron, copper, manganese, and zinc. Pollen composition is highly variable and depends on both the floral and geographical origins (De-Melo et al., 2018; Gardana et al., 2018; Liolios et al., 2019; Taha, 2015). The following trends in the elemental composition of pollen have been observed: major elements: P > K > Ca > Mg > Na, minor elements: Fe > Mn > Zn > Cu.

**Fig. 4:**
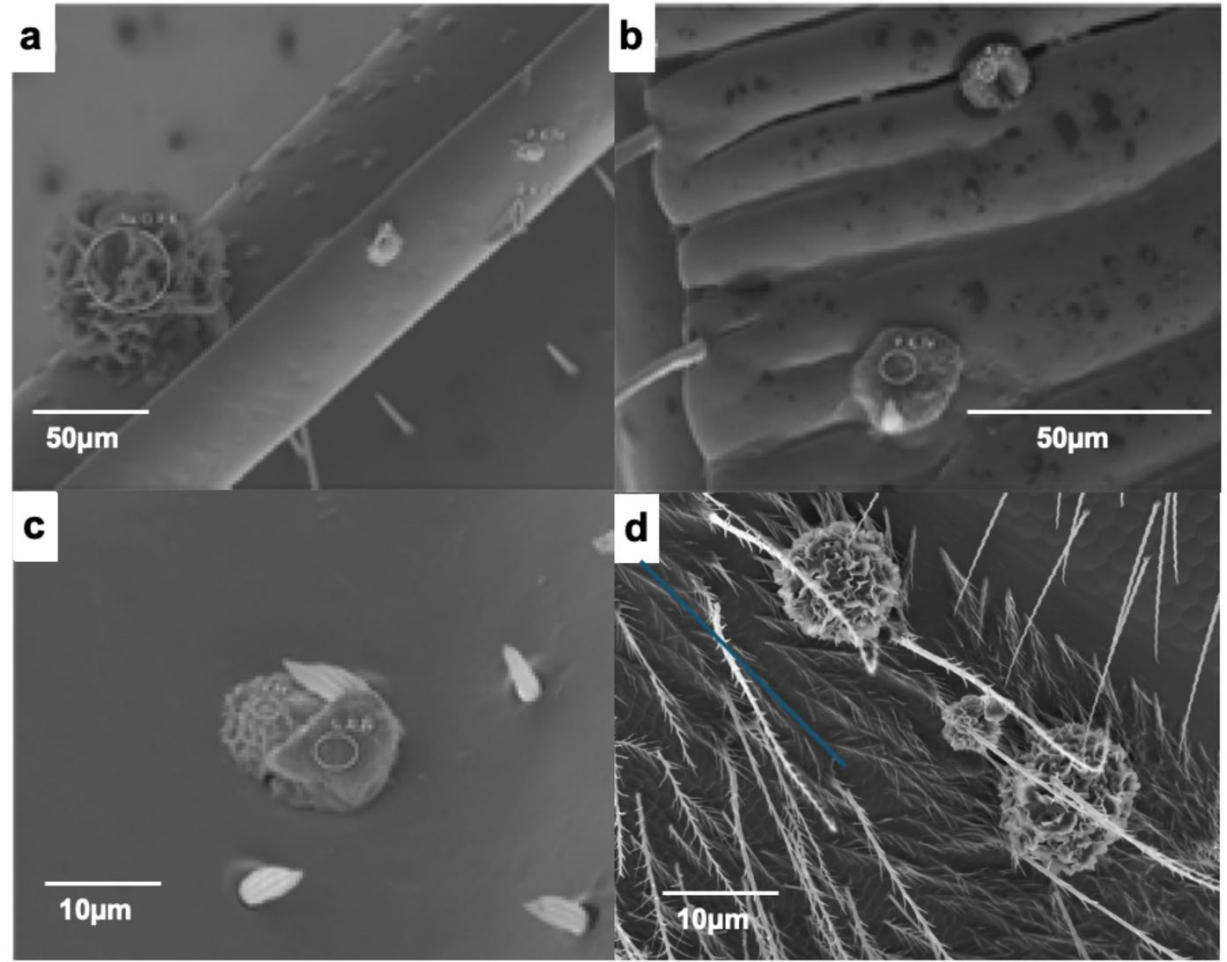
SE Images of gold-coated bees from Düdingen **a**, **c**, **d** and St. Silvester **b**. Often other particles are attached to the pollen, like a metallic iron fragment (**b** bright triangular patch at the bottom of the lower pollen) or an iron-containing mineral, **c** flake on the top of the pollen, possibly chlorite)

Minor peaks of K, P, and S are thus typical fingerprints for pollen spectra, and the corresponding subclass was classified as "pollen". Often, Fe and Cl were also present in the pollen spectra. Fe is a minor component of pollen. Cl is possibly originating from fertilizers. The main and most distinguishing characteristics of pollen particles are their distinct shapes, which are easy to image if electron charging is not too strong (Figs. 4a–d). Morphology was therefore also taken as a classification criterion.

### Minerals

**Fig. 5:**
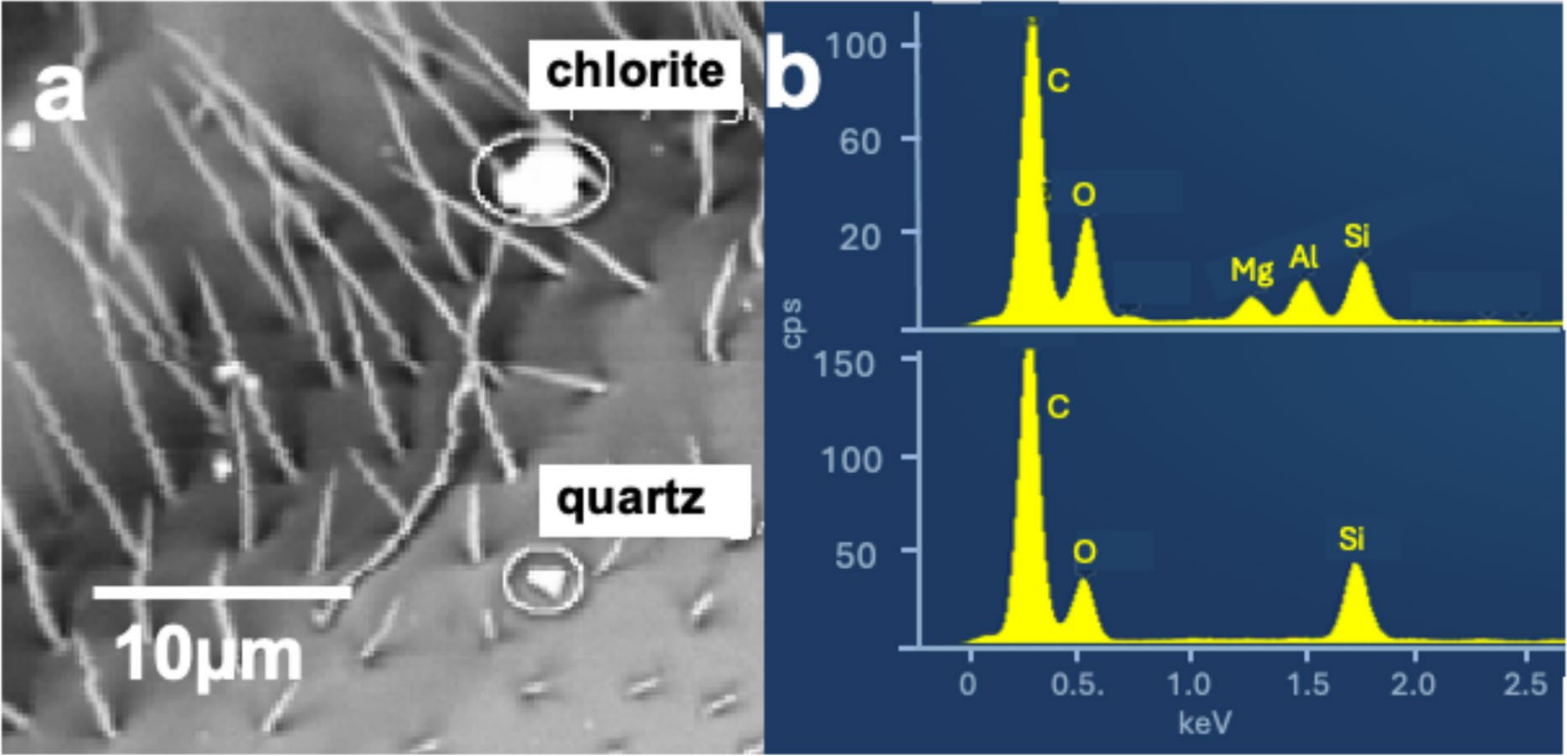
**a** BSE image and **b** corresponding EDS spectrum of a typical mineral-metal aggregate on the eye of a bee from the Plasselb apiary. The white spikes are scopae (body hairs). The main phases in the mixture are calcite (CaCO_3_) and titanium metal or oxide

**Fig. 6:**
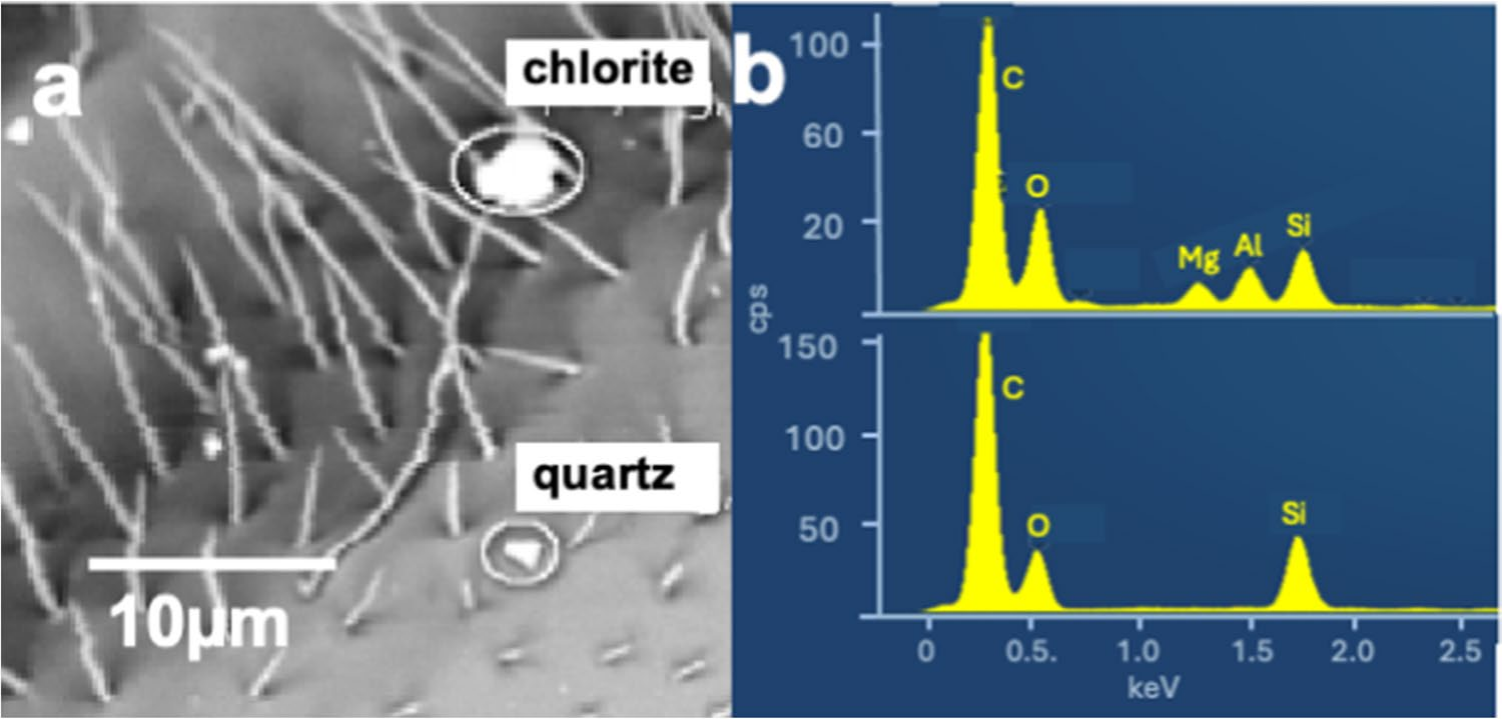
**a** BSE image of a wing of a DUD bee with two mineral particles and **b** corresponding EDS spectra

Mineral particles are related to the weathering of the rocks that dominate the geology surrounding the apiaries, e.g., sand-, lime-, and claystones of the Swiss Molasse Basin and the Prealps (Masson, 1976). The rocks in both regions consist of limestone composed of calcite (CaCO3; subclass (sc): Ca) (Figs. 5a, b). and/or dolomite CaMg(CO_3_)_2_ (sc: CaMg), sandstone containing quartz SiO_2_ (sc: Si, Figs. 6a, b) with minor potassium- and natrium feldspars (K, Na)(AlSi_3_O_8_) (sc: KNaAlSi), shales containing micas such as muscovite KAl_2_(AlSi_3_)O_10_(OH)_2_, biotite K(Mg,Fe^2+^)_3_(AlSi_3_)O_10_(OH)_2_ (sc: KMgFeAlSi), chlorite (Mg,Fe _6_(Al, Si)_4_O_10_(OH)_8_ (sc: MgFeAlSi, Figs. 6a, b) and kaolinite Al_2_Si_2_O_5_(OH)_4_ (sc: Al,Si). The "Mineral"-class particles contain mainly the elements Si, Al, K, Mg, Fe, and Ca. All subclasses representing partial combinations of these elements, except the subclasses containing either Fe or Al alone, were classified as minerals. The latter two were classified as metals, although they may also be oxides, carbonates, or hydroxides.

### Metals

**Fig. 7:**
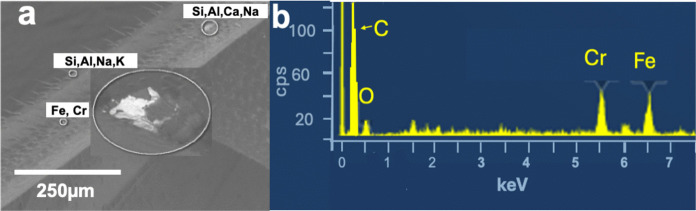
**a** Right wing edge of a Plasselb bee with two mineral particles (Si, Al, Na, K: kalifeldspar; Si, Ca, Na, Al: plagioclase) and a steel particle (inset: enlarged image of the latter), **b** corresponding EDS spectrum of the steel particle

EDS analyses of particles containing metallic elements have been recorded on all bees and filters. They may occur as pure metal particles, as oxides, or as components of more complex minerals, such as biotite. In the latter case, they are always accompanied by Si and Al. Cr-, Ni-, Co-, Mn-, Cu-, and Zn-containing silicates, carbonates, oxides, or sulfides in the rocks of the targeted area are very rare. The single particles or aggregates containing the latter metals are, therefore, most likely of anthropogenic origin. They are the result, for example, of metal processing in industry (Ahn & Lee, 2006; Marris et al., 2012) or of abrasion in the engines/brakes of cars or railway locomotives (Fussell et al., 2022; Thomas et al., 2024). Electric motors of appliances (Szymczak et al., 2007), and the friction between the locomotive pantograph and the catenary along railway lines produce Cu particles (Fruhwirt et al., 2023). Often Fe and Cr, as well as Cu and Zn, occur together, a typical fingerprint for steel (Fig. 7), respectively, brass. All the above-cited metals can also be present in gunshot residue (GSR) particles (see below). The heavy metals inhaled by, or attached to the bees from anthropogenic activities, are finally transferred to the beehives and possibly also to materials produced by the bees, such as honey and wax. This may pose a risk to the health of both bees and humans, who consume these products (Conti & Botre, 2001; Conti et al., 2022; Jomova et al., 2025; Kostic et al., 2020).

### Soot

**Fig. 8:**
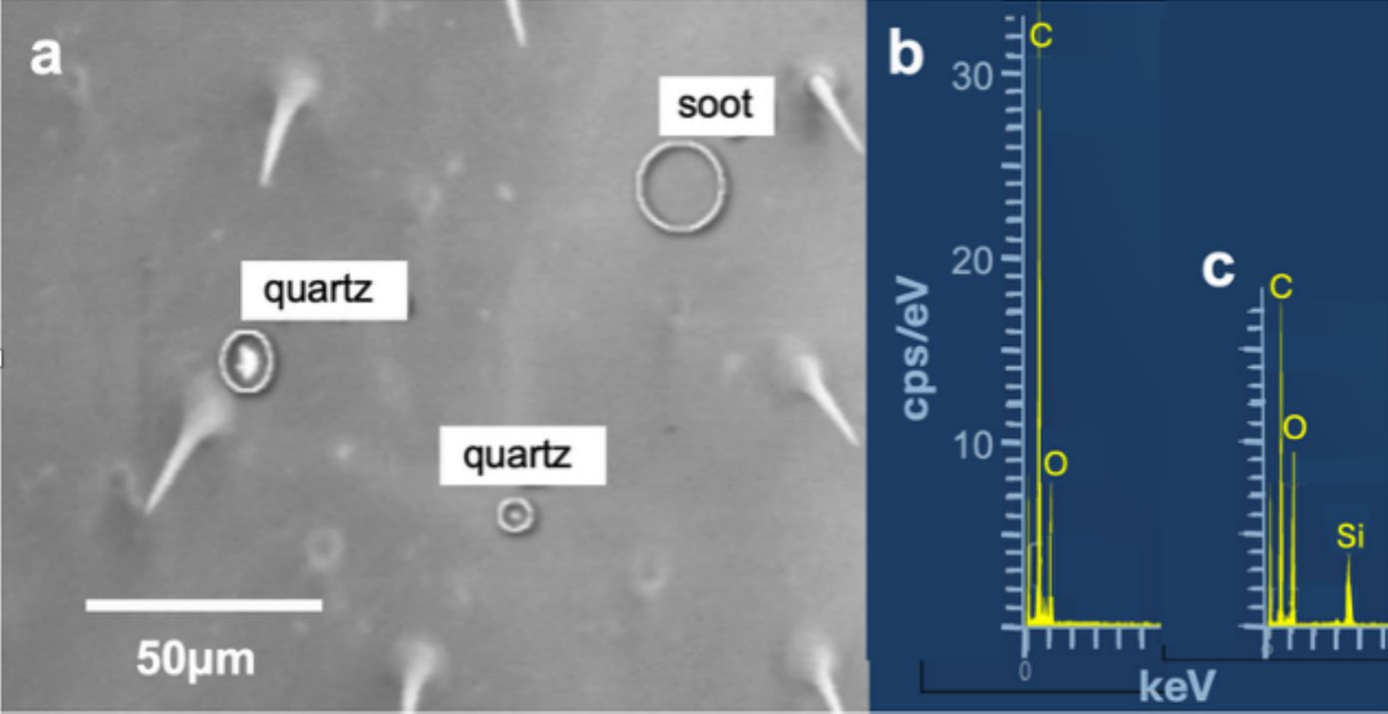
**a** SE image of the left wing of a DUD bee with quartz particles and a soot flake. **b**, **c** Corresponding EDS spectra. Note the twice as high carbon peak in the latter compared to the quartz spectrum. Both spectra were taken consecutively under the same conditions. In the quartz spectrum, the carbon intensity is only from the bee body and the coating

Soot particles have two main sources: fossil fuel combustion resulting from industrial activity, traffic, or heating, and wood burning. The soot particles found on the bees are typical of aged diesel soot (Medalia & Rivin, 1982). The primary diesel soot consists mainly of aciniform carbon, e.g., fractal-like dendritic agglomerates of nanosized carbon spheres. During transport, soot particles undergo modifications, a process known as aging (Vernooij et al., 2009; Weingartner et al., 1997). Aciniform soot undergoes restructuring and shrinking to a more compact form, which then grows again, especially under high relative humidity, low supersaturations, lower temperatures (Eriksson et al., 2017; Yuan et al., 2019) and in the presence of H_2_SO_4_ (Pei et al., 2018). There were no aciniform particles found on filters nor on the bee bodies, but only fragmental carbon particles (Fig. 8), also on the DUD bees with a beehive close to the highway (s. Figure 1). The meteorological conditions during campaign A in the Fribourg area, e.g., moderate average temperature of 10°, 80–100% relative humidity and a high atmospheric sulfate concentration (see below) were thus favorable for rapid transformation, which explains the presence of fragmental carbon particles.

### Salts

**Fig. 9:**
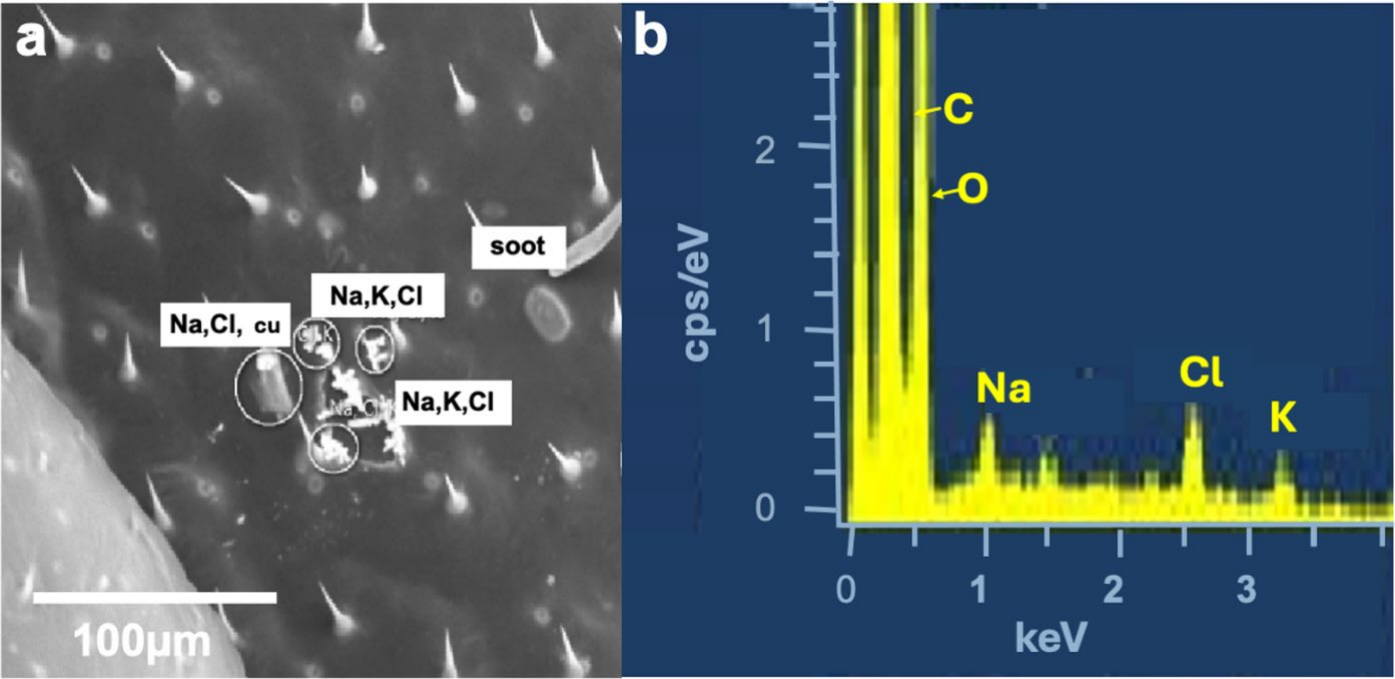
**a** Several dendritic salt aggregates on the right wing of a PLB bee containing Na, K, and Cl and one NaCl cube (cu), **b** corresponding EDS spectrum (the Al peak is from stray radiation of the in-lens detector)

Alkali chloride salts ((Na, K, Ca)Cl) (called "salt" in the following) were found on all filters and bees from all beehives. They are relatively rare on bees and appear as dendrites or crystal cubes (Figs. 9a, b). Salt can have either a natural or anthropogenic origin (Minguillón et al., 2012). They are emitted in the atmosphere over the ocean through sea spray or in desert regions as dust components. In Switzerland, salt particles of probable oceanic or desertic (Saharan Dust Emissions) origin have been detected in aerosol samples collected at the Jungfraujoch research station (Hinz et al., 2005). In inhabited regions of Switzerland, the main source of salt in the local aerosol is its use for winter road maintenance (Zuber et al., 2015), or in agriculture (DüV, 2024). Swiss de-icing agents are composed of sodium chloride, calcium chloride or magnesium chloride, urea, biodegradable lower alcohols, sodium and potassium formate and acetate, as well as molasses, but no KCl.

For the salt dendrites (Fig. 9a) on the bees, de-icing salt is rather unlikely because all chemical compositions of the latter contain potassium (Fig. 9b), as well as for the locations of some beehives, such as STS, the roads and trails within the bees’ foraging territory are cleaned mechanically during wintertime, and no de-icing salts are used. The dendritic and patchy shape of the salt is probably related to the bees’ dietary habits. Honeybees are known to prefer ‘dirty water’ that contains salts (Butler, 1940). Sodium, magnesium, and potassium are essential for the development of the larvae (Herbert & Shimanuki, 1978) and salts dissolved in water may, therefore, be a necessary part of the brood food (Brodschneider & Crailsheim, 2010). Bees can obtain salt from multiple sources, including carrion, feces, brackish water, and even human tears (Abrol et al., 2012; Banziger et al., 2009; Ferry & Corbet, 1996; Lau & Nieh, 2016). Honey bees appear to prefer agricultural or urban water runoff because it is abundant and contains high levels of salts. The PLB and STS beehives are in a region of heavy dairy farming – we are at the eastern boundary of the Gruyère cheese region! There are therefore many cattle feces, which also pollute the waterpools. The dendrites appear to have formed after the bees became wet while drinking from such brackish pools. If the elements Na, K, Mg, or Ca and Cl are present, the particles have been classified in the "Salt" group.

### Sulfates, nitrates

**Fig. 10:**
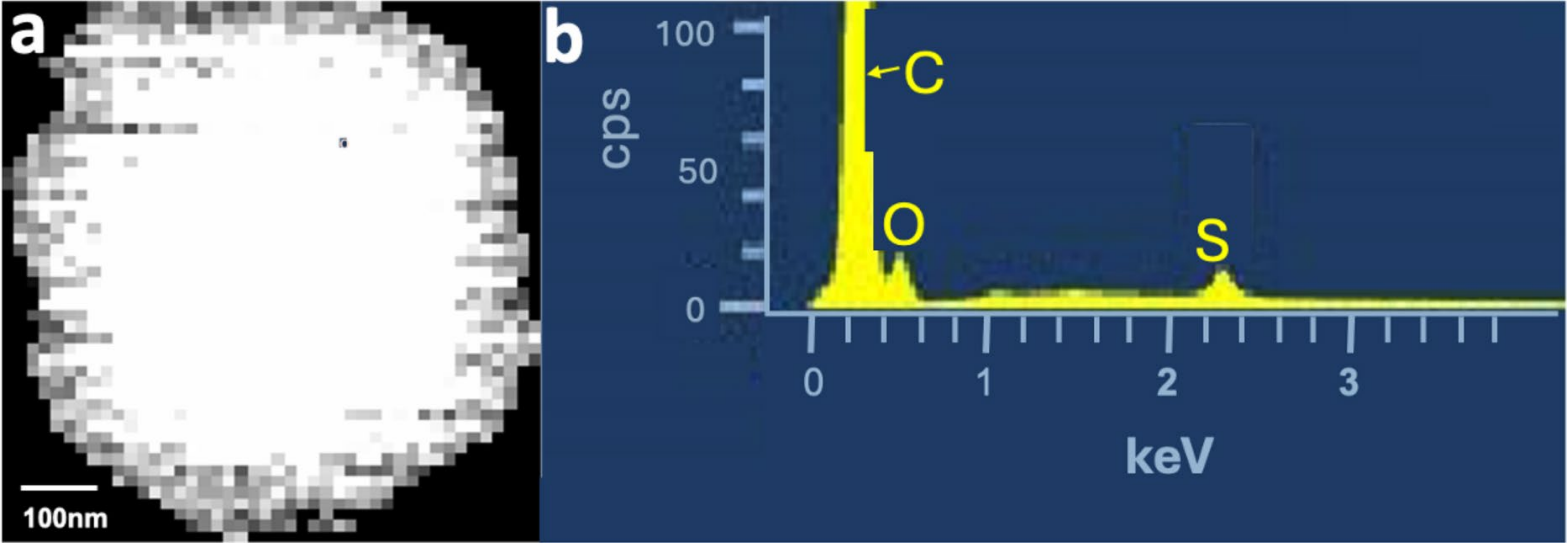
**a** BSE image of a typical sulfur-rich particle present on all filters, **b** corresponding EDS spectrum

Sulfur-rich particles, containing oxygen, without (Fig. 10) and with alkalis, were found on the filters of both campaigns, but not on a single bee. Sulfates are soluble, which may explain their absence in the bees. Salt, which is also soluble, is present on the bees but is rare. Salt-rich waters, in contrast to sulfate containing, are targeted by the bees. The highest concentrations were found on filters from PLB and JB. The most common forms of aerosols are ammonium and alkali sulfates, as well as H_2_SO_4_ (Tomasi & Lupi, 2017). The sulfate precursor is primarily SO_2_, which can originate from natural sources (e.g., volcanic gases) or anthropogenic activities (e.g., fuel combustion, cattle feces). H_2_SO_4_ droplets are produced through oxidation of SO_2_ by OH° radicals, which is made in sunlight (Wang et al., 2006). Evaporation residues of H_2_SO_4_ sampled in clouds (Ganor, 1999), captured in aircraft exhaust (Pueschel et al., 1998) or artificially produced (Worobiec et al., 2003), have similar morphologies and EDS spectra as the sulfur and oxygen containing particles (all around 100 nm in size) on our sampling surfaces. Ambient nitrate and sulfate in urban and rural areas act as sinks for ammonia, with sources including animal waste, fertilizer application, soil release, and industrial emissions (Rietra et al., 2024). In most European countries, winter restrictions for the application of manure to agricultural fields have been lifted (DüV, 2024; Font et al., 2024). The Gruyère region has a significant dairy farming presence, which explains the high concentrations of sulfate, ammonium, and nitrate in the rural regions of the canton of Fribourg (e.g., measurements at the aerological center of Meteoswiss in rural Payerne, 17 km west of Fribourg (Hüglin & Grange, 2021)). Alkali sulfates are typical transformation products of salt in sulfate-rich atmospheres (Adachi & Buseck, 2015), which is consistent with the presence of NaCl in all samples. Atmospheric ammonium nitrate aerosol forms when sulfate is fully neutralized and ammonia (NH3) is in excess (Hartmann et al., 2013). Nitric acid (HNO_3_), a precursor formed together with ammonia, is emitted by heavy emitters of NO_x_ (Wei et al., 2023). Not surprisingly, sulfate-containing aerosols are always dominant in the analyzed samples, given the sites’ location away from heavy NO_x_ emitters.

### Gunshot Residues GSR

**Fig. 11:**
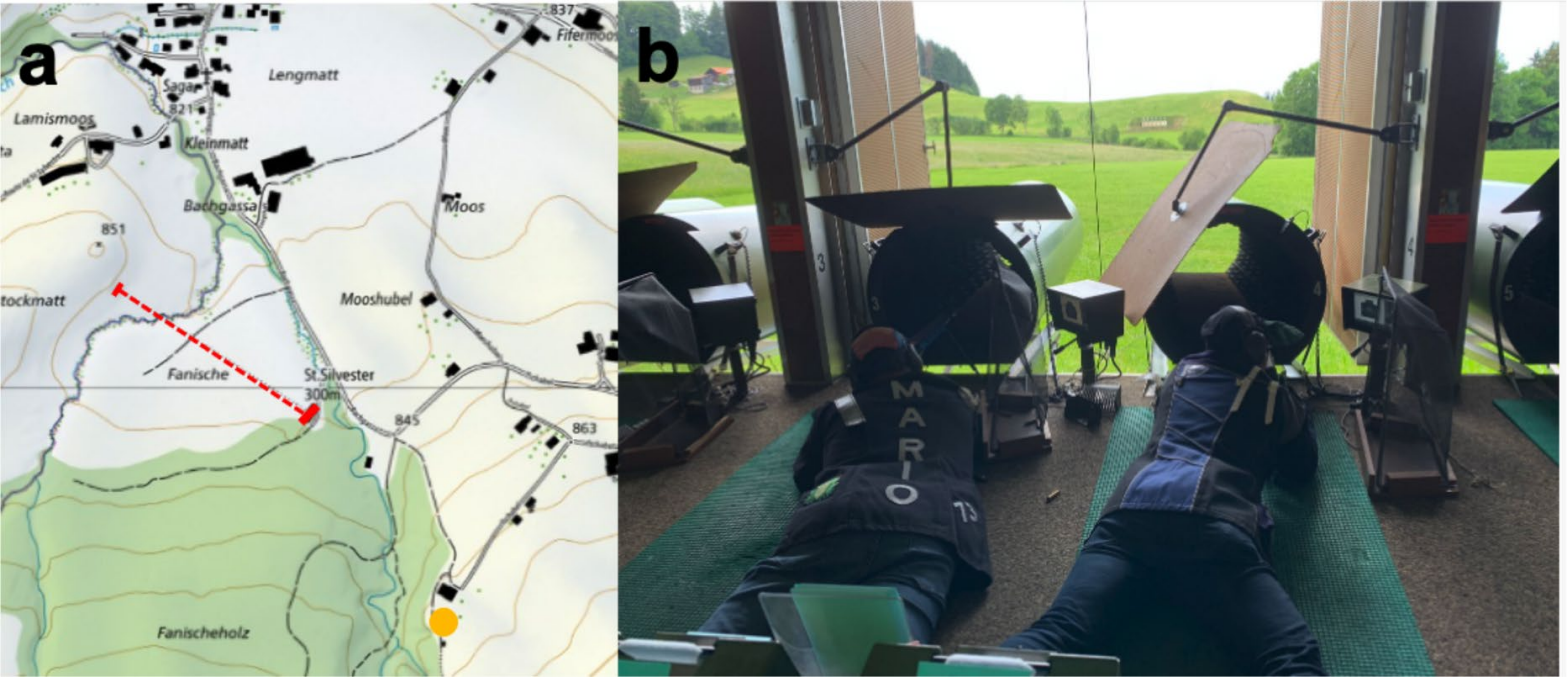
**a** The shooting range (red) is 400 m from the STS beehive (orange point), separated by a small wood (https://www.swisstopo.admin.ch). **b** Six stands shooting range of St Silvester (https://www.sgstsilvester.ch/fotogalerie-1/2022/)

**Fig. 12:**
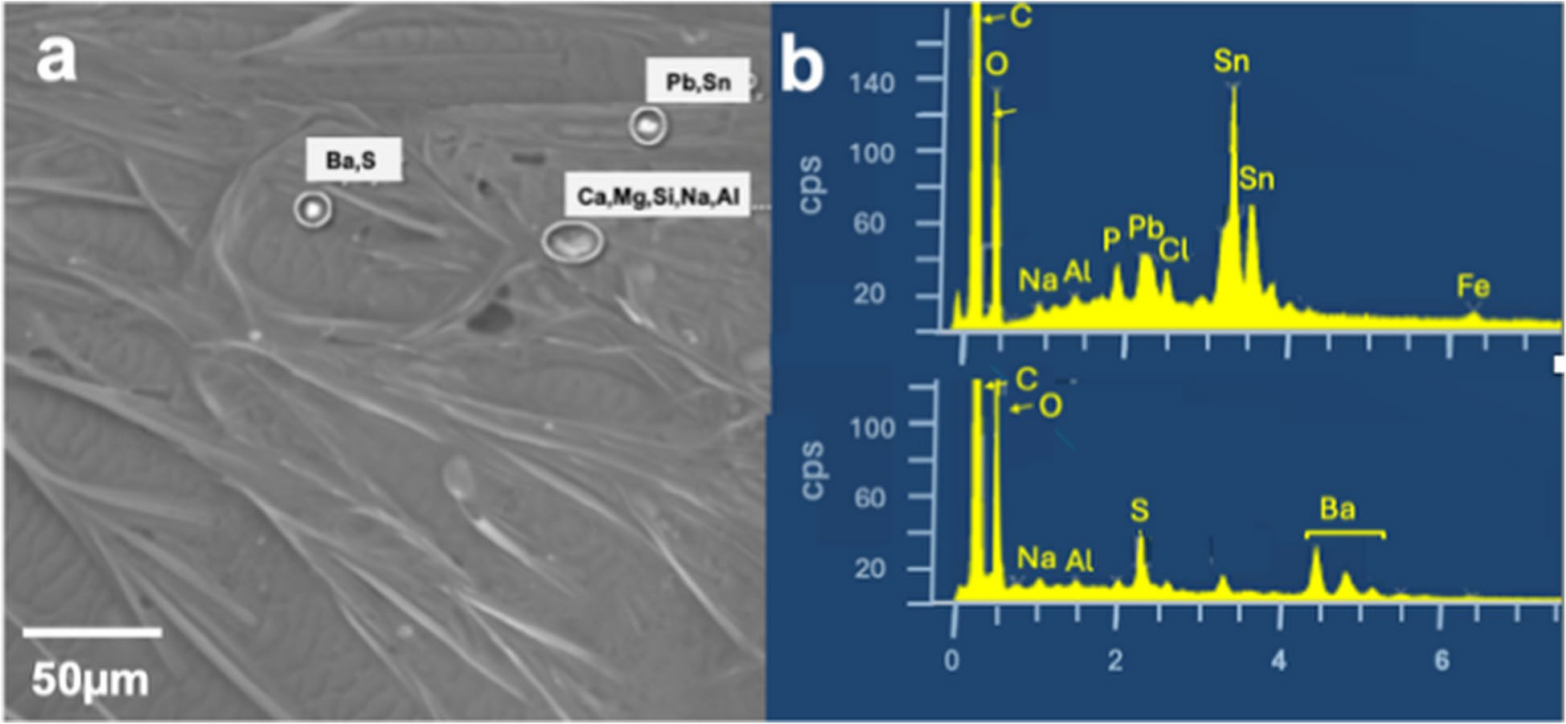
**a** SE image taken from one of the front legs of a St. Silvester bee. Among the particles, there are two items with GSR typical elements (Pb, Sn, and Ba, S) and a mixture of dolomite (CaMg(CO_3_)_2_) and albite (NaAlSi_3_O_8_). **b** EDS spectra of the GSR particles

**Fig. 13:**
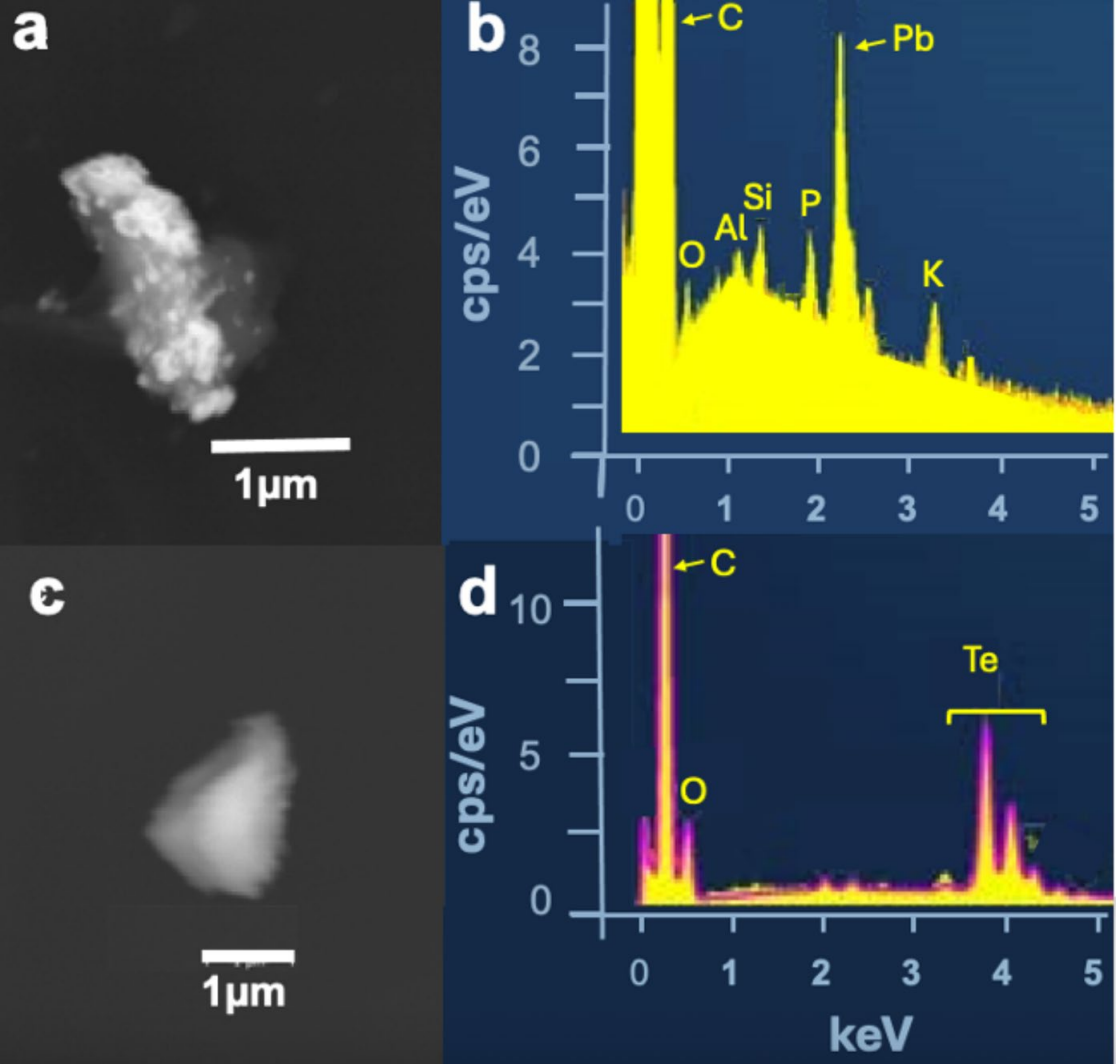
**a** Particles found on STS bees: **a** lead particle, the white flakes on the surface are most likely micas as indicated by K and Al peaks in the corresponding EDS spectrum **b**. **c** Almost pure tellurium particle and **d** corresponding EDS spectrum

On bees collected at St. Silvester beehive particles with rather unusual elements such as Ba, Pb, Sb, Hg, Cr, Ni, Cu, Zn, and Sn (Figs. 11a–c, 12a–d) were detected, which are highly toxic already in small quantities and count as pollutants (Masindi & Muedi, 2018). Sources for this element profile are uncommon, even in industrial settings, but it is typical of gunshot residues (GSR) originating from the gun or the cartridge (Kim et al., 2022). Modern gun cartridges are composed of the casing, the bullet, the propellant, and the primer, which is typically a shock-sensitive chemical that ignites the latter. The propellant is the most important organic contribution of GSR. The traditional propellant, black powder, has been replaced in modern ammunition by nitrocellulose, nitroglycerin, and nitroguanidine, used in various proportions, along with lubricants, plasticizers, stabilizers, and other additives in small quantities. Inorganic particles are emitted from the primer, from the bullet, and from the gun barrel itself. Inorganic elements and compounds in ammunition that may contribute to GSR (Dalby et al., 2010): Casings of cartridges are made of Sb, Bi, Ni, and P, bullets contain elements such as Sb, Cr, Pb, and Ti. The primer mix (initiating explosives, oxidizing agents, fuel, and sensitizers) contains Al_2_S_3_, Sb_2_S_3_, Sb_2_(SO_4_)_3_, Ba(NO_3_)_2_, BaO_2_, B, CaSi_2_, CuSCN, Pb(N_3_)_2_, PbO_2_, Pb(NO_3_)_2_, C_6_HN_3_O_8_Pb, Pb(SCN)_2_, Mg, Hg, Hg(CNO)_2_, KClO_3_, Si, NaNO_3_, Sr(NO_3_)_2_, S, Sn, Ti, ZnO_2_ or Zr. Particles from the barrel are composed of Fe and Cr, while particles from the cartridge, casing, and bullets contain Al, Fe, Cr, Cu, Zn, Bi, and Sn. Ammunition manufacturers have begun introducing lead-free primers (Heavy Metal Free, HMF), thereby reducing Pb exposure (Thomas, 2019). Cu and Zn particle concentrations, however, remain high also with non-Pb ammunition. The GSR composition depends on the type of gun being fired: whereas PM from handguns has high levels of Pb, rifle smoke has high levels of Cu (Kim et al., 2022). The reason for this difference is the mechanical action (e.g., friction and velocity) of the bullet on the barrel (Grabinski et al., 2017). The ignition of the primer and, subsequently, of the propellant induces two sharp increases in pressure and temperature, leading to the vaporization and decomposition of the primer compounds (Wolten & Nesbitt, 1980). After leaving the barrel, the vaporized material cools rapidly, condensing into liquid droplets and eventually forming solid particles that join the already solid metal particles. During the flight, non-GSR particles collide and stick with the GSR particles (Fig. 13a), resulting in agglomerates with lead, antimony, or barium oxides or sulfides (primer particles), as well as different lead, tin, zinc, and copper alloys cores (bullet and its jacket, cartridge or casing particles). The overall particle size distribution near the barrel is bimodal, with two maxima: one at approximately 0.3 µm, corresponding mainly to primer particles, and the second at around 1 µm, corresponding to bullet particles. Both peaks are related to the formation processes of the smoke, with the accumulation mode (small particles) associated with combustion processes and the coarse mode particles generated by mechanical processes. Sizes of GSR particles found on the STS bees belong to both modes.

Handgun GSR particles can be detected up to 18 m from the position of the shooter, even 24 h after the shot has been fired (Luten et al., 2018). For GSR from a machine gun (like the Swiss soldiers’ gun) fired outdoors with a longer barrel compared to a handgun, this distance is probably larger, considering that atmospheric turbulence may transport the particles.

There is indeed a 6-stand 300 m shooting range, separated by a small wood, at a distance of 500 m from the St. Silvester apiary (Figs. 11a, b). The meadows separating the shooting stands from the targets contain flowering plants and represent, therefore, food sources within the foraging area for the St. Silvester bees. The local shooting club uses the shooting range. The shooting period begins in March and typically has 3 to 10 official shooting days per month. In early June, there is an intense shooting schedule, mainly due to the annual military service duty, which is mandatory for every Swiss man.

The filter samples of Campaign B were taken before and after this period. Different weapons of the Swiss army are usually used, which can be loaded with both lead-containing or HMF ammunition. The most commonly used ammunition is the 7.62 × 51 mm NATO/GP 11 cartridge. Most types of this ammunition have a lead core, SINOXIDE primer (DYNAMIT NOBEL AG) composed of lead tricinate, tetracene, barium nitrate, lead dioxide, antimony trisulfide, and calcium silicide, and a low zinc-copper alloy jacket (called "tombac"). The ammunition used by the sports shooter is not specified. The elements Ba, Cu. Zn, Cu, Sn, and Pb are dominant in GSR particles on STS bees (Figs. 12a, b, 13a, b). The almost pure tellurium particle found on a filter indicates that HMF ammunition (Figs. 13c, d) (Flint & Gombar, 2016) is also used in St. Silvester. Particles with GSR typical elements were found very rarely on filters, but not on bees from apiaries other than STS. 

### Particle concentrations

#### Filters

**Fig. 14:**
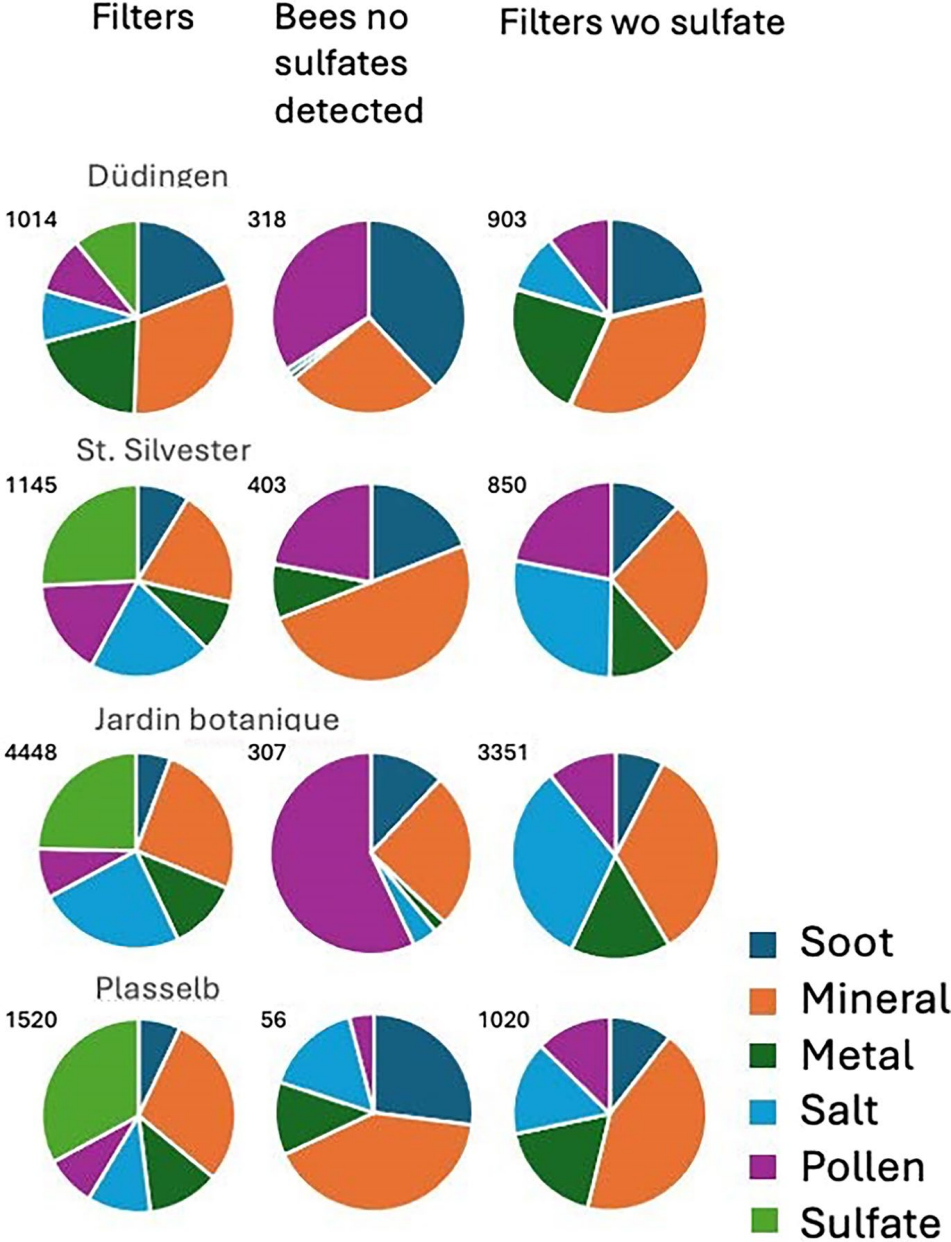
Proportional distribution of the six particle groups on bees and filters in the four sites in campaign A. The last column shows the distribution on filters, excluding sulfates (and nitrates). On the upper left of each pie chart, the number of analyzed particles is indicated

For the filter collection, the sampling period, duration, sampled volume, and location are precisely known (see Table 1), whereas these parameters for bees as particle monitors are unknown. In contrast to particle sampling by foraging bees, each air sample collects aerosols transported to a single location over a limited time span. These samples depend on the strength and direction of the prevailing winds, which may introduce a strong bias in the sources contributing to the sample. The particle-emitting sources are not necessarily next to the beehive, and the heavier and bigger particles of such distant sources may not arrive at the filter sampling location. An advantage of the air sampling measurement is the homogeneity of the polycarbonate filter, which is protected from the environmental conditions during sampling. It features an even surface with a homogeneous background, enabling automated particle analysis with AZTec. Therefore, particle analyses on filters are quantitative, whereas those on bees are not. Analyzing particles on bees provides better insight into possible sources in the near field, e.g., the bees’ foraging territory, independent of wind direction. The particle types and concentrations on the bees do not necessarily have to match those collected on the filters. To compare concentrations between filters and bees, the six filter groups were reduced to five, eliminating the sulphate group, which was not present on bees (Fig. 14).

##### Campaign A

Because the number of particles differ very much from sample to sample we report the proportional contribution of each group to the overall count of particles (Fig. 14). On the filters mineral and sulfate (and nitrates) particles have the highest proportions underlining the rural environment (even for the botanical garden) of the sites and diary farming as main agricultural branch. Not surprisingly, soot and metal particle concentrations are highest in the vicinity of the highway and the railway tracks in Düdingen. Campaign A occurred at the end of September and the beginning of October, when atmospheric pollen concentrations for all plant species in Switzerland are low (Gehrig & Schwierz, 2024; Riessberger & Crailsheim, 1997) and the proportion of pollen on the filters was similarly low. The salt proportion is highest at the Jardin botanique site and astonishingly lowest at the Düdingen site, close to the highway, where de-icing salts are used during winter. However, sampling occurred long after the last use of de-icing salt, which may explain the low salt concentration.

##### Campaign B, St. Silvester

**Fig. 15:**
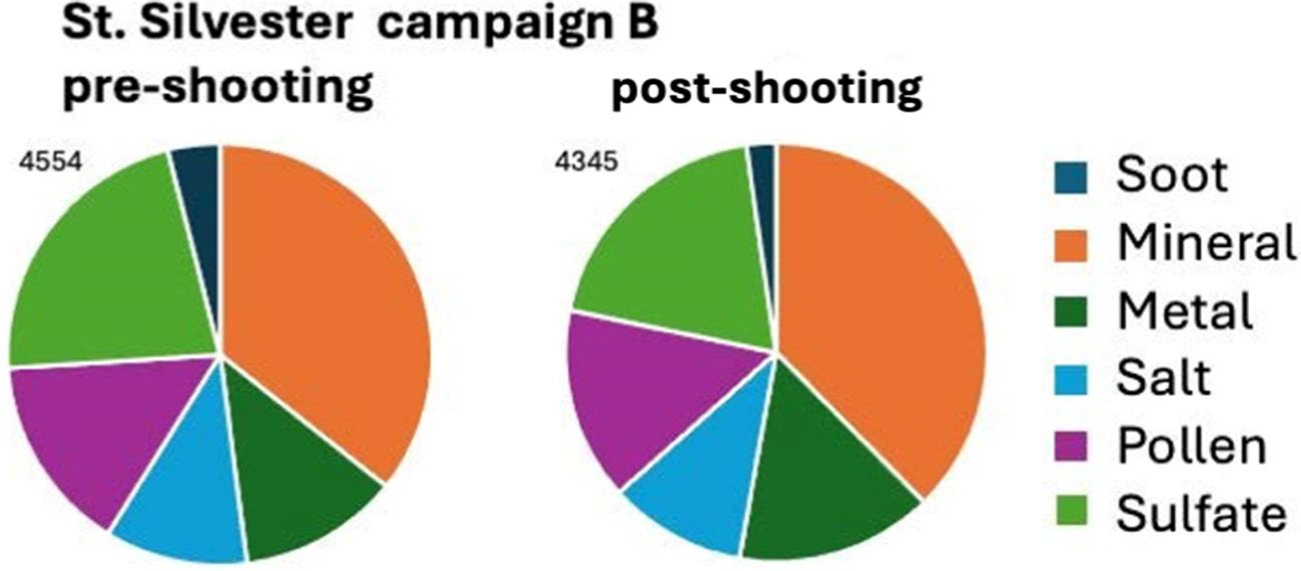
Proportional distribution of the six classes on filters in St. Silvester during campaign B

Although the filters were taken during late springtime and at a different place in St. Silvester compared to campaign A (shooting range vs. beehive), both samples taken before and after the intense shooting period have a similar aerosol profile as the sample taken in autumn (Fig. 15). The pollen contribution is slightly higher which is to be expected, considering that it was flowering season. However, it was also towards the end of the hay harvest, which explains why the concentration was not even higher. The difference between the pre- and post-shooting is a slightly higher metal concentration (661 vs. 553 particles), explained by the additional GSR metal particles (292 vs. 219 particles, Fig. 15).

### Bees

#### Campaign A

Particles from the 6 groups present on the filters, except sulfate group particles, were also found on all bees (Fig. 13). The salt and metal proportions, except for the PLB bees, are very low. Bees require water for their own hydration, to maintain osmotic balance, to prepare liquid food for the brood, and to cool the hive on hot days (Winston, 1987). Foragers collect water from streams, ponds, and other wet areas (Kühnholz & Seeley, 1997). Although the bees’ bodies are hydrophobic due to the cuticular hairs with a thin layer of lipoid material on the outer surface of the tips (Jones, 1955), wings are not hydrophobic (Bello et al., 2022). Bees often fly close to the water surface. When falling into the water, their buoyant bodies provide flotation, but the wings get wet. They are unable to free their wings from the water surface, but by beating them, they transform into hydrofoils, producing forward thrust that lifts them out of the water (Roh & Gharib, 2019). The partly hydophobic nature of the bee’s body, its water-collection habit, and encounters with light rain are probably the reasons for the scarcity of soluble salt and sulfate particles on the bees’ bodies. The salt dendrites and patches were found on the hydrophilic wings and are probably the result of the drying of a bee after contact with salt-containing water just before being collected.

The scarcity of metal particles found on DUD and JB bees is possibly related to a selective removal of such particles. It is well known that certain elements, especially metals and metalloids, have a detrimental impact on bees (Gekiere et al., 2023). To investigate their environment, bees rely on visual, olfactory, chemotactile, gustatory, and even electric information (Nicholls & Ibarra, 2017). Bees acquire the corresponding information through numerous receptors located in sensilla found on antennae, mouthparts (proboscis), and tarsi (Chapman, 1998). Experiments testing the capacity of antennae and proboscis receptors to recognize certain elements dissolved in aqueous and sucrose solutions have shown that honeybees are sensitive to these elements, but the sensitivity varies significantly with their chemical identities and concentrations as well as the body parts in contact with them (Al Naggar, 2017; Al Naggar et al., 2020; Burden et al., 2019; Di et al., 2016; Hladun et al., 2012; Monchanin et al., 2022; Søvik et al., 2015). Burden et al. (2019) showed that Cu was rejected following antennal but not proboscis stimulation. Monchanin et al. (2022) demonstrated that As, Pb, and Zn were rejected when their antennae or proboscis were stimulated; however, Cd had no effect. However, this was true only for high, non-field-realistic concentrations, as confirmed by electrophysiological analyses. The toxicity of low concentrations of Al, Cd, and Pb was tested (Gauthier et al., 2016). In recent years, there have also been a series of publications on the recognition of nanoparticles (NPs) by bees and the effect they have on them (Al Naggar, 2017; Kos et al., 2017; Liu et al., 2021; Ozkan et al., 2014; Viana et al., 2025). Most of the investigations focused on the toxicity of NPs to bees. A few two-choice experiments were conducted, presenting NP-free, pure sucrose solutions and NP-loaded sucrose suspensions to the bees. Even though zinc salts are mostly deterrents to bees (Burden et al., 2019), bees prefer the highest concentrations of ZnO NPs tested (Glavan et al., 2017). Although this is contrary to the results with Zn^+^-solutions, it suggests that bees can detect nanoparticles. A possible detection mechanism for ZnO NPs is through the distinctive mechanical properties of ZnO NP suspensions. The only investigation that examined the difference in effects, but not in detectability, between nano and non-nano particles was conducted on boron particles (Dağlioğlu & Kabakçi, 2015). No experiments have been conducted to date to test whether bees can recognize the composition of particles deposited on a solid surface. The receptors identified so far in honeybee gustatory sensilla are primarily devoted to sugar perception (Sanchez, 2011). All these results thus do not exclude the possibility that bees can chemically distinguish particles on their bodies and (selectively) remove them during social grooming and self-cleaning.

#### Campaign B

**Fig. 16:**
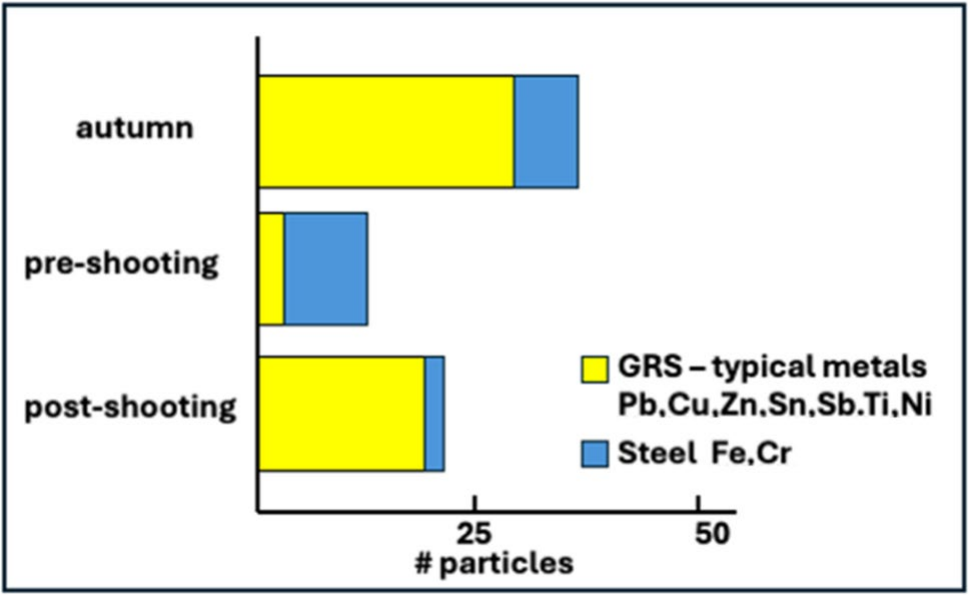
Metal particles found on the bees of the St.Silvester

GSR particles were found on the STS bees, independent of the campaign. The lowest number of GSR particles was found in bees collected in spring, before the intense shooting period (Fig. 16). Unexpectedly, the highest number was observed not after the intense shooting week, but in autumn. During the summer, shooting continues, but GSR particles introduced into the atmosphere during this period will be washed out by wet deposition. Most likely, the particles have been transferred to the bee’s body by contact with vegetation.

## Statistical analyses

Descriptive statistical analyses (Table 3) were performed on the autumn bee results. The nonparametric Kruskal*–*Wallis test (Table 4) was used to examine whether there were any mean differences in the number concentration (NC) of the five particle groups on bees from the four beehives with JASP (JASP_Team, 2025). The normality of particle NCs was assessed using the Shapiro–Wilk test. There were no outliers identified on the different NCs in the different beehives, except for the mineral and salt NCs in the JB beehive. We decided to retain these outliers, given the small sample size, to avoid removing datapoints from the model. The NCs were normally distributed across all beehives, as skewness and kurtosis statistics fell within the acceptable range of −2 and + 2, except for the pollen kurtosis (2.652). The Q-Q plots of the soot and pollen variables show linearity in the middle of the Q-Q plot, but curve off at the extremities. The homogeneity of variances was not met, as assessed by Levene’s test for equality of variances (*p* > 0.05), except for the pollen type (*p* < 0.05). The presence of outliers and heteroscedasticity led us to use the nonparametric Kruskal*–*Wallis test (Table 4) instead of ANOVA. The Kruskal–Wallis test shows that there is no significant difference among the bees for any of the variables, and Dunn’s post hoc test shows no significant pairwise differences.

**Table 3:** Descriptive statistics for the five particle types on the 16 bees of the five hives

XX	BEES	*N*	Mean	SD	SE	CV
Minerals	DUD	4	28.50	9.256	4.628	0.325
	JB	4	30.75	6.946	3.473	0.226
	PLP	4	36.00	22.524	11.262	0.626
	STS	4	44.00	6.377	3.189	0.145
Metals	DUD	4	8.750	6.850	3.425	0.783
	JB	4	9.500	4.203	2.102	0.442
	PLP	4	7.250	7.762	3.881	1.071
	STS	4	19.250	2.500	1.250	0.130
Salt	DUD	4	8.750	6.850	3.425	0.783
	JB	4	9.500	4.203	2.102	0.442
	PLP	4	7.250	7.762	3.881	1.071
	STS	4	19.250	2.500	1.250	0.130
Pollen	DUD	4	12.750	7.890	3.945	0.619
	JB	4	14.250	3.304	1.652	0.232
	PLP	4	27.750	22.794	11.397	0.821
	STS	4	7.000	1.826	0.913	0.261
Soot	DUD	4	34.50	28.688	14.344	0.832
	JB	4	25.25	9.946	4.973	0.394
	PLP	4	24.25	22.559	11.280	0.930
	STS	4	12.75	3.775	1.887	0.296

**Table 4:** Kruskal–Wallis Test

					95% CI for Rank ε^2^			95% CI for Rank η^2^	
Particle type	Statistic	df	*p*	Rank ε^2^	Lower	Upper	Rank η^2^	Lower	Upper
Minerals	3.916	3	.271	0.261	0.129	0.813	0.076	0.000	0.754
Metals	7.138	3	.068	0.476	0.320	0.879	0.345	0.156	0.857
Salt	8.670	3	.034	0.578	0.493	0.883	0.473	0.309	0.869
Pollen	3.739	3	.291	0.249	0.137	0.845	0.062	0.000	0.832
Soot	2.262	3	.520	0.151	0.050	0.710	0.000	0.000	0.670

## Honey analyses

We analysed honeys from three beehives (Table 5) to see if the heavy metal pollution translates into the heavy metal content of the honey. The claims in the literature are controversial. Whereas some authors consider that honey is a good indicator of environmental contamination by heavy elements/minerals (Bartha et al., 2020; Bogdanov, 2006; Crane, 1984; Ruschioni et al., 2013), others have the view that only pollen, propolis, wax, and bees themselves are valid indicators of environmental heavy metal content but not honey (Borsuk et al., 2021; Conti & Botre, 2001). Many authors have shown that bees act as "biofilters" during the transformation of nectar into honey, purifying the latter by removing these contaminants (Conti et al., 2018, 2022; Dżugan et al., 2018; Losfeld et al., 2014; Tomczyk et al., 2020). Also, in heavily contaminated areas (e.g., mining and industrial areas), honey meets the residue limits for contamination with heavy elements/minerals (Erbilir & Erdoĝrul, 2005). Analysed bee bodies have higher contamination levels than corresponding honey (Bogdanov, 2006). Reduction factors are impressive: honey contained 40-fold lower Fe, 26-fold lower Zn, and eightfold lower Cu and Cd. Pb is only reduced fourfold (Borsuk et al., 2021). Except for the amount necessary for the bees’ metabolism (Kuterbach & Walcott, 1986; Nichol & Locke, 1995), the excess amounts were excreted in feces (Skalny, 2014).

**Table 5:** Analyses of honey samples. The values are given as mg/kg of honey

beehives	Sb	Ba	Cu	Pb	Zn	Hg
JB	nd	0.122	1.38	0.022	0.738	nd
STS	nd	0.127	0.249	nd	0.981	nd
PLB	nd	0.127	0.211	nd	0.838	nd

*nd*, below detection limit

The heavy element contents in the three analysed honeys (Table 5) were below the limits tolerated by the European Commission (EC, 2013, 2015, 2023), which is also valid in Switzerland. For honey, the maximum limits in mg/kg are: Pb, 0.10 mg/kg; Cu, 0.5 mg/kg; Zn, 1.5 mg/kg; Fe, 7.0 mg/kg. Except for copper in the JB sample, which is above the limit (1.38mg/kg), all measured elements in the three analysed honeys are below the limits. The STS results are consistent with the fact that, for example, the transfer of heavy metals into the corresponding honey is not observed.

## Conclusions

Aerosol sampling was conducted in early summer and late autumn in the canton of Fribourg (Switzerland) using active sampling and particles collected from bees. Particle collectors were placed in the vicinity of 4 beehives situated in an urban and three rural locations. The particles found on the filters reflected typical environmental sources of the locations. Mineral particles are predominant on all filters. Higher proportions of soot and metal were found near a highway and adjacent railway line. The high salt content, despite the limited use of de-icing salts and sulfates, can be attributed to an essential economic sector in the Canton of Fribourg: dairy farming. The sampling after the hay harvest of the first campaign and the autumnal second campaign resulted in a relatively low proportion of pollen. The bees had the same types of particles on their bodies, but with a different distribution than on the filters, except for sulfate and nitrate particles, which were lacking on all bees. The GSR particles on the St. Silvester bee were found to originate from a 500 m distant shooting range. All elements typical for gunshot residues (Ba, Cu, Zn, Sn, Pb, etc.) could be detected and confirmed by the air sampling measurements next to the shooter’s cabin. Therefore, bees represent a valuable tool for detecting potential sources of particulate matter emissions, especially to discover small emitters with rather "exotic" particle compositions. Except for the GSR, no alarming PMs were found. Analyzing particles on honeybee body surfaces with the Scanning Electron Microscope is an interesting tool for exploring the particle burden in the local atmosphere. Although the qualitative advantage of analyzing PM on bees is evident, a quantitative analysis is not possible because bees clean themselves, change their foraging area, and not every part of a bee can be appropriately analyzed with SEM (nor with CT scanning).

## Data Availability

No datasets were generated or analysed during the current study.
